# The NKF-NUS hemodialysis trial protocol - a randomized controlled trial to determine the effectiveness of a self management intervention for hemodialysis patients

**DOI:** 10.1186/1471-2369-12-4

**Published:** 2011-01-28

**Authors:** Konstadina Griva, Nandakumar Mooppil, Penny Seet, Deby Sarojiuy Pala Krishnan, Hayley James, Stanton P Newman

**Affiliations:** 1 Department of Psychology, National University of Singapore, 9 Arts Link AS402/28, Singapore; 2 National Kidney Foundation, Kim Keat Road, Singapore; 3 Unit of Behavioural Medicine, Riding House Street, University College London, UK; 4 Health Services Research Group, City University, College Building Room A224 St John Street, London, UK

## Abstract

**Background:**

Poor adherence to treatment is common in patients on hemodialysis which may increase risk for poor clinical outcomes and mortality. Self management interventions have been shown to be effective in improving compliance in other chronic populations. The aim of this trial is to evaluate the effectiveness of a recently developed group based self management intervention for hemodialysis patients compared to standard care.

**Methods/Design:**

This is a multicentre parallel arm block randomized controlled trial (RCT) of a four session group self management intervention for hemodialysis patients delivered by health care professionals compared to standard care. A total of 176 consenting adults maintained on hemodialysis for a minimum of 6 months will be randomized to receive the self management intervention or standard care. Primary outcomes are biochemical markers of clinical status and adherence. Secondary outcomes include general health related quality of life, disease-specific quality of life, mood, self efficacy and self-reported adherence. Outcomes will be measured at baseline, immediately post-intervention and at 3 and 9 months post-intervention by an independent assessor and analysed on intention to treat principles with linear mixed-effects models across all time points. A qualitative component will examine which aspects of program participants found particularly useful and any barriers to change.

**Discussion:**

The NKF-NUS intervention builds upon previous research emphasizing the importance of empowering patients in taking control of their treatment management. The trial design addresses weaknesses of previous research by use of an adequate sample size to detect clinically significant changes in biochemical markers, recruitment of a sufficiently large representative sample, a theory based intervention and careful assessment of both clinical and psychological endpoints at various follow up points. Inclusion of multiple dependent variables allows us to assess the broader impact on the intervention including both hard end points as well as patient reported outcomes. This program, if found to be effective, has the potential to be implemented within the existing renal services delivery model in Singapore, particularly as this is being delivered by health care professionals already working with hemodialysis patients in these settings who are specifically trained in facilitating self management in renal patients.

**Trial registration:**

Current Controlled Trials ISRTN31434033

## Background

End stage renal disease (ESRD) is a complex disease associated with compromised quality of life (QoL), unplanned hospital admissions, high mortality and therefore high burden of illness [1,2]. With rising prevalence worldwide, the growth of ESRD populations has been a concern for many countries, as ESRD consumes increasing proportions of healthcare budgets [3,4]. The burden of ESRD will increase with the growth of the ageing population [5,6] and increased prevalence of diabetes [7].

As in other countries the incidence and prevalence of ESRD in Singapore is rising [7]. The incidence rate has inflated from 194.0 per million population (pmp) to 284.9 pmp from 1999 to 2007 [8]. This increase has been standardized for age differences between the population cohorts and hence is an underestimate as there is an escalation in the elderly population of Singapore. Singapore has one of the fastest aging populations in the world. When elderly is defined as a person of 65 years of age and above, the number of elderly has expanded from 47,200 in 1965 to 330,000 in 2009 [9]. This rapid expansion of the elderly in Singapore will have a significant effect on rates of ESRD.

Mortality is high among patients receiving renal replacement therapy with an 8% mortality rate in the first 90 days [10]. In Singapore, survival during the first year of dialysis is 89.3% and 58.7% after a period of 5 years [9]. Risk factors for mortality include older age, physical and nutritional impairment, smoking, prior myocardial infarction, low serum albumin levels at baseline, catheter access at first dialysis, concomitant cancer, heart failure, depression, HIV/AIDS, lung disease, neurological disease, psychiatric conditions and late referral to a Nephrologist [11,12].

Cardiovascular disease (CVD) accounts for approximately 50% of all deaths [13]. Dialysis patients are on average at approximately 30 times higher risk of a fatal cardiovascular event than the general population, and this risk still remains 10 to 20 times higher after stratification for age, gender and diabetes [13]. This burden of CVD has been associated with both traditional risk factors such as smoking, diabetes, hyperlipidaemia, hypertension and physical inactivity and non-traditional chronic kidney disease-associated factors such as anaemia, hyperhomocystenaemia, oxidative stress and bioincompatibility [14]. Several investigators have identified associations between elevated serum phosphate, calcium × phosphate product, parathyroid hormone and death in hemodialysis patients [15]. Interventions that lower serum phosphate levels and thereby delay the development of secondary hyperparathyroidism may reduce cardiovascular mortality.

Hemodialysis requires radical lifestyle changes including regular attendance at the dialysis unit for treatment, restrictions in fluid intake, changes to diet and medication intake. Estimates within the haemodialysis population suggest that the prevalence of non-adherence is between 10% to 60% for fluid intake, 2% and 57% for dietary advice, between 0 and 35% skip or shorten dialysis sessions and between 19% and 99% are non-adherent to their medications [16]. This is likely to be associated with psychosocial variables such as patients' beliefs about medication, social support, and personality characteristics rather than clinical or sociodemographic variables [17,18]. Poor adherence can have a significant impact on the risk of morbidity and mortality. In one large scale study, when compared to compliant patients, those who skipped one or more hemodialysis sessions in a month had 5.7% greater interdialytic weight gain and higher serum phosphate levels. Those who had shortened the duration of 3 or more of their hemodialysis sessions over the period of one month had a between a 13% and 35% higher risk of death [19]. It has been estimated that each 1-mg/dL increment in phosphate level increases relative and cardiovascular mortality risk by 5% and 10% respectively [20,21]. Especially when phosphate levels are > 5.5-6.0 mg/dL, mortality risks start to increase considerably [21,22].

Self management interventions offer an effective tool to support adjustments to the lifestyle changes required in hemodialysis. For self management to be effective, it needs to encompass the patient's ability to monitor their condition and to affect the cognitive, behavioural and emotional responses necessary to maintain a satisfactory QoL [23]. There are 5 core self managements skills; problem solving, decision making, resource utilization, forming of a patient/heath care provider partnership and taking action [24].

Although there is evidence to suggest both clinical and psychological benefits resulting from self management interventions in chronic conditions such as arthritis, diabetes and recurrent lower urinary tract infections [25-27], there is comparatively less work on the value of self management programs in renal disease.

Cross sectional research within the Chronic Kidney Disease (CKD) population [28] and with kidney transplant patients [29] has shown that perceived self efficacy is a more consistant correlate of the performance of self management behaviours than clinical and demographic variables. In addition engagement in self management behaviours has been associated with improved QoL [30]. There have however been very few randomised controlled trials assessing the impact of self management in hemodialysis. Adaptation training and patient empowerment programs with patients on hemodialysis have been shown to reduce perceived stress, depression and improve self care self-efficacy and QoL at 3 to 6 months post intervention [31-33]. A group based intervention using cognitive behavioural techniques to enhance effective self management of fluid consumption has also been shown to improve interdialytic weight gains although these effects were evident only at 14 weeks follow up [34]. Although these studies suggest that psychosocial interventions increase adherence and improve success in self-care in the context of hemodialysis, most have been underpowered, the interventions are poorly described, follow up duration is limited and rigorous evaluation through the inclusion both clinical and psychological outcomes data are lacking. Reviews have also criticized these studies for not describing in detail the required training, content or theoretical background for the intervention and intervention delivery and fidelity [35-38].

The proposed study will address weaknesses of previous research by recruitment of a sufficiently large and representative hemodialysis sample, the development of a well designed theory driven and evidence based intervention and the inclusion of validated assessments of both clinical and psychological outcomes up to 9 months post intervention. In doing so this trial will provide much needed data on the efficacy of self management interventions with respect to clinical and psychological outcomes that can inform clinical practice and health care services.

This paper describes the design, setting, intervention and outcomes of the NKF-NUS program, a 36 month cluster randomised trial of self-management support program for prevalent hemodialysis patients.

### Study Objectives

1. To implement a group based self management intervention for patients on hemodialysis

2. To undertake an evaluation of this self management intervention comparing it with "standard care". The primary aim is to determine the efficacy of the self management intervention on biochemical markers of clinical status and adherence. Secondary aims are to determine the effect impact on QoL, mood, self-efficacy and patient satisfaction.

## Methods/Design

### Trial design

**Figure 1:**
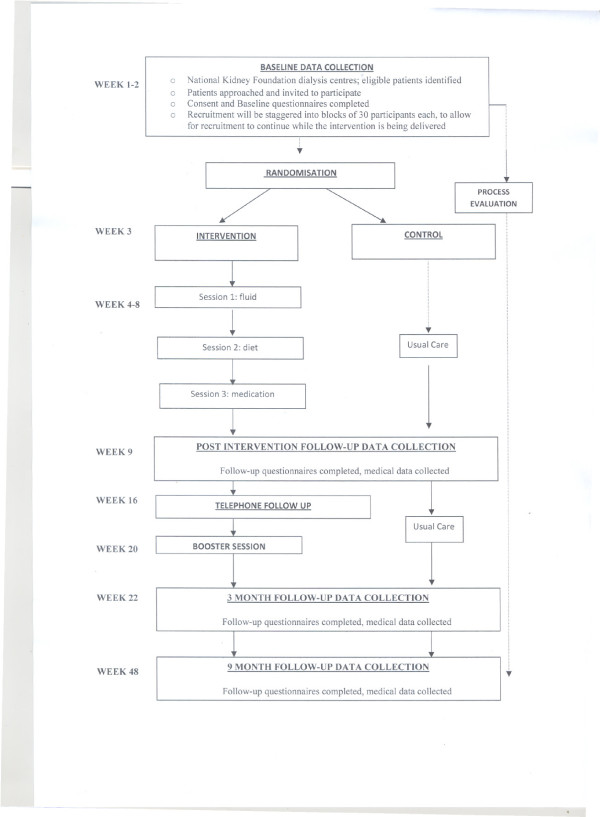
**Flow Chart of Study Design**.

The study design (Figure 1) is guided by the CONSORT statement [39]. The NKF-NUS Study is a 3 year pragmatic cluster randomized clinical trial to evaluate whether hemodialysis patients benefit from a self management intervention. The study protocol has been approved by the NUS Institutional Review Board and is in compliance with the Helsinki declaration.

Participants will be randomized based on dialysis shift within each of the participating NKF dialysis centres into one of two groups;

Group 1: Standard care control group - care currently received by patients as defined by the National Kidney Foundations Clinical Practice Guidelines. All healthcare resources used and advice given to prevalent patients relating to their kidney failure and its treatment will be standardised.

Group 2: Group based self management intervention

Patients from both arms will be assessed with the same measures and at the same time intervals over the study period.

### Setting and Centre Recruitment

The study will be conducted at the National Kidney Foundation (NKF) dialysis centers, Singapore. There are 24 NKF dialysis centres in Singapore providing hemodialysis to more than 2100 ESRD patients. These are located throughout the island to ensure easy community access to patients residing in different parts of Singapore. NKF Dialysis centres are nurse led with a multidisciplinary team of renal consultants, medical social workers, dieticians and exercise specialists on rotation.

Of these 14 NKF dialysis centres will be included in the program. Although random selection of dialysis centres would have been preferable this was not an option due to the lack of facilities in their excluded dialysis centres to either host the intervention in situ or their considerable distance to other dialysis centres with available facilities. Location and proximity to intervention venues was an important parameter to take into account as the intervention sessions are scheduled over weekends and hence require substantial commitment on the part of patients and dialysis centre staff.

### Participants

The senior nurse manager in each of participating dialysis centres, will screen patients for eligibility and provide a list of eligible patients and their preferred language of communication to the research coordinator. A research assistant (RA) proficient in the patients' preferred language of communication (e.g. English, Mandarin, or Malay) will then approach eligible patients for participation. Participants will be given an information sheet outlining the details of the study and the RA will subsequently verbally explain the study procedures and requirements, and provide clarifications as needed.

Following written informed consent, participants will be requested to undertake a baseline assessment (Time 1) (comprising the self report questionnaires listed in the following section) prior to group allocation (intervention vs. control). Follow up assessments will be taken immediately after the end of self management intervention (Time 2) and at 3 months (Time 3) and 9 months post intervention (Time 4) (See Figure 1).

Inclusion criteria are:

(1) Chronic Kidney Disease patients who have been receiving hemodialysis for at least 6 months

(2) Aged 21 and over

(3) Patients willing to attend all sessions of the self management programme.

Exclusion criteria are:

(1) Newly established on hemodialysis (< 6 months)

(2) Unable to give informed consent

(3) Unable to understand spoken English and/or Mandarin, Malay, Tamil dialects to allow effective communication with the intervention facilitator(s) and/or Research assistants

(4) A diagnosis of functional psychosis or organic brain disorder

(5) Impaired cognition

(6) Major visual or hearing impairments, or other sensory or motor impairments that may prohibit completion of the scheduled assessments

(7) Unable to participate in a group program (e.g. housebound)

(8) Limited life expectancy due to co-morbid illness such as malignancy

### Randomization

Cluster randomization will take place whereby patients within the participating dialysis units will be randomised according to the days on which they receive dialysis. For example, within a centre those dialysing on Monday, Wednesday and Friday will receive the intervention and those dialysing on Tuesday, Thursday and Saturday will serve as controls. This will prevent cross contamination of information between groups, a problem encountered when studying participants who spend large amounts of their time together in a closed environment.

Once a block of 30 participants have provided informed consent and completed baseline assessment, randomization will be performed by the study coordinator, using a computerized method to avoid assignment bias. Due to the nature of the study and in common of studies if this type, patients will not be blinded to their group allocation. Primary outcome measures will be assessed by a research associate blinded to treatment allocation and uninvolved in consenting and the management of the patients. Health care professionals and dialysis personnel involved in patients' care will also be blind to group allocation. It is not however deemed possible to keep project staff tracking data collection for secondary outcomes (self reported outcomes) blinded to condition so the trial will only have double blind accuracy with respect to primary outcomes. However, as questionnaire assessments are by self-report, rather than rated by a member of the research team, significant influences of observer biases are not expected.

### Participant withdrawal of consent to research follow-up

If a participant withdraws their consent whilst in the trial, one of the study team will contact the participant to determine if they are willing for the data they have given up to that point to be included in the trial. No data will be used in the analysis without a participant's consent.

### Sample calculation

Using serum phosphate levels as a physiological index of adherence to diet and medication) and Kidney disease specific QoL as primary outcomes, a total sample size of 126 and 128 respectively, were calculated. This was using 80% power (alpha = 0.05) to detect a significant absolute change of 1.5% in phosphate control and of 5 points (0.5 standard deviation) in the physical and mental component subscale scores in kidney disease QoL short form questionnaire [40]. Taking the larger number of 128 participants (64 in intervention and 64 in control group), a final total number of 154 participants (77 in each group) is needed to ensure at least an 80% final response rate is met (attrition/drop-out rate estimated at 20%). A target sample of 154 participants will be sought.

### Measures

The following demographic information will be collected before the start of the trial: age, gender, ethnicity, education, religious affiliation, marital and employment status, perceived work ability and household income, length of time since diagnosis, prescribed drugs currently taken, and co-morbid medical conditions.

#### Primary outcome measures

Beginning at baseline (Time 1) and continuing into follow up (Time 2, Time 3, Time 4) regular chart reviews will be conducted to abstract clinical, laboratory, and pharmacological data including:

(1) Interdialytic weight gain: The amount of weight gained between the end of one dialysis session and the beginning of the next. This is a validated marker of adherence to fluid restriction/regulation.

(2) Blood pressure

(3) Serum phosphate and calcium × phosphate product indicating a patient's adherence to prescribed oral phosphate binder medications and dietary phosphate restriction.

(4) Serum potassium indicating adherence to dietary recommendations.

#### Other Clinical Measures

(1) Medical notes will be also reviewed to record other serological data (urea, creatinine, hemoglobin, intact parathyroid hormone and albumin as well as urea reduction ratio and Kt/V), all prescribed medication and relevant information regarding dialysis history and dialysis related events (e.g. access complications) during the study period.

(2) Attendance for dialysis (skipping and shortening behaviours) and Health services utilization (number of admissions, emergency room visits) will also be monitored.

(3) The End Stage Renal Disease Severity Index (ESRD-SI) [41] will provide a measure of co-morbid illnesses and other complications of ESRD.

(4) The Charlson Comorbid Index (CCI) will be used to consolidate comorbidity burden. CCI scores will be computed pursuant to the methods described by Beddhu et al. [42].

#### Secondary process and outcome measures

The potential benefits of the intervention are quite broad, so multiple secondary outcomes are of interest and will therefore be measured with the following self report questionnaires. License agreements have been obtained for all measures. When no standardized translations of the instruments are available, translation validity will be confirmed using forward-backward method.

(1) QoL as assessed using the Kidney Disease QoL Short Form - KDQoL-SF [43]. This measure contains the Short form 36 health survey questionnaire (SF-36) supplemented with scales targeted at particular concerns of individuals with kidney disease: symptoms, effects of kidney disease on daily life, burden of kidney disease, cognitive function, work status, sexual function, quality of social interaction and sleep. To minimize burden of completion the SF 36 of the scale has been replaced with the SF12, a shortened validated version of SF36 to gather information on eight health concepts: physical functioning, role limitations due to physical and emotional health, mental health, bodily pain, general health, vitality and social functioning. These items are then scored using a norm-based method providing a component summary scale score for both mental and physical Health Related Quality of Life [44]. The instrument has been proved reliable and valid in dialysis patient populations in Singapore [45].

(2) Generic/Global quality of life was assessed using the *World Health Organization Quality of Life *questionnaire (WHOQOL-BREF) [46]. It is a self-report inventory of generic QoL consisting of 26 original items. The items fall into four domains: a) *Physical Health*, b) *Psychological Health*, c) *Social Relationships *and d) *Environment*, while two items provide a measurement of an *Overall QoL/Health *facet. The scale has demonstrated good psychometric properties. It is rated on a 5-point Likert scale and the range of scores is between 1-20 with higher scores indicating better quality of life.

(3) Anxiety and depression according to the Hospital Anxiety and Depression Scale (HADS) [47]. This is a widely used, self-administered questionnaire specifically developed to detect anxiety and depression states in hospital and medical out-patient clinic settings with good reliability and responsiveness [48]. It is composed of two 7-item scales, one for anxiety and one for depression. The original English version has been translated into and validated in many languages, including Chinese [49,50].

(4) Self-efficacy will be assessed using 2 measures: one is specific to dialysis patients and the self efficacy for managing chronic disease scale [51]. The latter comprised the sum of self-efficacy for managing the general demands of chronic disease. Respondents rated their confidence for performing various chronic disease self-management tasks including, seeking information about their conditions, obtaining help from others and communicating with physicians, maintaining role function, and managing symptoms.

The dialysis specific Self-Efficacy Scale [27] was devised specifically for the purposes of this study. Respondents are asked to indicate their level of confidence in managing dialysis specific demands related to diet, fluid intake and medication using eight items scored on 10 point likert rating scale ranging from 'not all confident' to 'totally confident' in line with the Self efficacy for managing chronic disease scale [51]. An expert panel of renal health professionals has reviewed the items and the scale was successfully piloted with a small group of hemodialysis patients (N = 4).

(5) The Health Education Impact Questionnaire (HEIQ™) is a reliable measure with high construct validity, designed to evaluate outcomes from patient education and self-management interventions for people with chronic conditions. It comprises eight domains to assess more proximal program outcomes [52]; Health directed behaviour, Positive and active engagement in life, Emotional well-being, Self monitoring and insight, Constructive attitudes and approaches, Skill and technique acquisition, Social integration and support, Health service navigation.

(6) The Renal Adherence Attitudes Questionnaire (RAAQ) [53] - The RAAQ is a 26-item scale measuring general attitudes toward compliance. The scale is composed of Likert-type statements, which measure a patient's attitudes toward social restrictions, well being, self-care/support and acceptance.

(7) The Renal Adherence Behaviour Questionnaire (RABQ) [53]- The RABQ comprises 25 items measuring self-reported dietary and fluid intake compliance. Specific dimensions; include compliance to fluid restrictions; compliance regarding potassium and phosphate restrictions, compliance regarding self-care; compliance regarding sodium intake; and compliance in times of particular difficulty.

(8) Beliefs about medication [54]. The measure comprises two 5-item sub-scales assessing beliefs about the necessity of prescribed medication ('necessity' sub-scale) and concerns about prescribed medication based on beliefs about the danger of dependence and long-term toxicity and the disruptive effects of medication ('concerns' sub-scale).

(9) The Medication Adherence Report Scale (MARS). The five-item MARS asks respondents to rate the frequency with which they engage in non adherent behaviors (e.g., deciding to miss a dose, forgetting to take a dose). Scores for each of the five items are summed to give a total score ranging from 5 to 25 where higher scores indicate higher levels of self-reported adherence [55].

(10) Six items developed for the study will be included to measure frequency of non adherent behaviours to dietary recommendations (3 items) and fluid intake (3 items) (e.g. forgetting, adjusting).

(11) Qualitative assessment

The trial also includes a qualitative sub-study. The qualitative research involves in-depth interviews of intervention participants at 3 and 9 months after delivery of the NKF-NUS self management course. The aim of this work is to explore participants' attitudes towards the program, their satisfaction with content, delivery and duration and their progress with regards to self management.

Participants will be asked to describe whether they have tried making any changes in their fluid intake, diet and general lifestyle or thinking as a result of the intervention; whether these changes have been made successfully or unsuccessfully and the reasons why. Participants will also be asked to highlight the strengths and weaknesses of the intervention and to give feedback on the program in five open-ended questions.

A short intervention evaluation questionnaire developed for the purposes of the study will also be administered at the 3 and 9 month follow up to rate patients' satisfaction with the content, length, delivery of individual sessions, dynamics and interaction with the facilitators and with other patients in the group.

### NKF- NUS Hemodialysis Self management Intervention

The NKF NUS hemodialysis self management program is based on the UCL diabetes self management program [27,56] but adapted to meet the needs of the target hemodialysis population in the local context. It emphasises patients' central role and responsibility in managing their illness. The program offers the potential for people to learn about their condition and treatment in a psychologically motivating and confidence enhancing structure - emphasis is on empowering patients to make choices and lifestyle changes in line with treatment recommendations through the use of problem solving, goal setting and feedback.

The NKF-NUS hemodialysis self management program has been developed after widespread consultation with patients and health care professionals using focus groups and in depths interviews.

The group based intervention consists of three main sessions held every two weeks and one booster session (a total of four 90 minute long sessions each with a 15 minute refreshment break halfway through). Sessions are facilitated by two health care professionals with experience of working with dialysis patients (medical social worker, renal nurse, renal dietician and/or psychologist). This has been planned to ensure assimilation of the self management program into existing renal services. Table 1 summarises program content.

**Table 1:** Summary of Content of the NKF-NUS self management program

Session	Topic	Content
1	Fluid intake	General introduction to program; expectations;
		Introduction to fluid regulation; barriers to fluid regulation; problem solving; goal setting on fluid intake
2	Diet	Feedback on progress
		Review and revise goals from session 1.
		Introduction to Healthy eating in the context of ESRD; Difficulties related to diet; problem solving, goal setting on diet
		+ BROCHURE 'Healthy eating for people of dialysis'
3	Medication *	Feedback on progress
		Review and revise goals on diet and fluid
		Introduce to medication for patients on dialysis; barriers to taking medication; problem solving; goal setting on medication.
		Discussing potential/preventing Goal Lapses
		Dealing with Goal Lapses
		+ BROCHURE 'process of problem solving'
	* Exercise [only if group has no issues with medication]	Introduce exercise for patients on dialysis; Barriers to exercise; problem solving' goal setting on exercise
	Telephone follow up	Feedback on progress
		Review and revise goals
4	Booster session	Group activity to revisit program topics; revisiting expectations
		Feedback on progress; Review and revise goals
		Use problem solving to overcome problems
		Lapses, relapses and maintenance of behaviour
		Maintaining changes over time
		Goal Setting
		+ BROCHURE 'process of problem solving'

Sessions in the program are based on psychological theories and techniques that have previously been employed to enhance and maintain health behavior change [57,58]. Intervention components will include problem solving, overcoming barriers, challenging beliefs, conducting brainstorming sessions, goal setting, and reinforcement and group processes. These self-management techniques are taught by means of skills mastery through biweekly action planning and feedback on progress, modeling of self-management behaviours and problem-solving strategies, and social persuasion through group support and guidance for individual self-management efforts. Targeted behaviours will include fluid control, diet, medication and/or exercise.

Patients are required to implement the coping-strategies taught from each session between sessions. Each session will be broadly structured to consist of a brief introduction to the theme, elicitation of patients' views on the topic, addressing of misconceptions, group discussion of possible coping strategies, identification of barriers to change, training in specific management strategies, drawing up of individual goals to be achieved, formulating actions plans to achieve these goals and reviewing previously set goals.

Patients will be contacted by telephone 2 months post intervention by one of the group facilitators (either psychologist, nurse or social worker) to assess the progress they are making with their goals. This will provide an ongoing interaction between the healthcare professional and the patients and will thereby represent an additional support system. A booster session will also be provided for participants 3 months after completion of the initial intervention. This will allow patients the opportunity to review their progress, address maintenance of behaviours over time and explore reasons for success and failure. The booster session will also remind patients of the principles of self management, problem-solving, action planning and will provide revision of important behavioural areas and reset goals. This booster session will be led by the same group facilitators.

### Missed sessions

If participants do not attend a session they will be sent any missed materials and, where possible, the session will be held over the telephone by one of the facilitators. Participants receiving the intervention will continue to have access to services available as part of their usual health care

### Delivery of intervention

To implement the proposed self management intervention, a number of healthcare professionals including renal nurses, renal dieticians and medical social workers already working extensively with the renal patient population in the National Kidney Foundation will be trained in the self management principles required for delivery of the intervention. This will allow the facilitators to run group sessions using the self management skills of problem solving, decision making, resource utilization, formation of patient/heath care provider partnerships and taking action. This program will be formulized in a manual with an accompanying training course, thereby facilitating rollout across the health/renal services and enabling any benefits accruing in the study to be broadly implemented.

### Quality assurance and Fidelity of Intervention

Fidelity rating for the intervention will first be evaluated in the preliminary phase of supervised pilot sessions; this consists of 4 pilot groups which will be scheduled to complete prior to the main trial to enable further training for facilitators. Intervention consistency and quality will also be assessed throughout the trial. A random subset of the intervention sessions will be observed. The delivery of the intervention will be evaluated using a criterion based checklist for each session. The criteria will include assessment of intervention techniques used (e.g. rapport building, problem solving, reinforcement) as well as a checklist against the content objectives for each session (e.g. whether facilitators kept to their roles and how well they explained/guided participants on setting goals). Summary notes from those facilitating the sessions will also be collated for further analysis. A sub-sample of the intervention facilitators will be asked to participate in a brief semi-structured interview to understand their experience of the intervention and their perceived competence in delivering and facilitating the session. Finally structured notes (e.g. how long each session, where it was delivered, etc.) will be collected to ascertain the effect of various setting and contextual factors.

A trial steering group will meet at regular intervals throughout the trial and advise on any issues that arise. The senior research team will provide feedback and support to the data collection and intervention facilitators' teams.

### Statistical Considerations

Outcomes will be analysed immediately after the intervention and then at 3 and 9 months

separately on an intention to treat basis. Analyses of covariance (ANCOVAs) will be performed for each outcome to examine changes within groups (Intervention vs. standard care control) comparing baseline to follow-up assessments. Covariates used will include baseline levels and other casemix differences between the groups (if any). The statistical significance criterion will be set at *P *< 0.05.

In addition to this intention-to-treat analysis we will also undertake a "per protocol" analysis on those individuals who attended all of the educational sessions in the intervention arm.

No formal subgroup analyses are planned. However exploratory analysis of the impact of patient level factors (e.g. age, gender, ethnicity and time on dialysis) on the effect of the intervention will be carried out.

### Data management

Each participant will be assigned a unique numeric study code at the beginning of the trial so that they can be tracked anonymously throughout. The trial data will be entered onto a SPSS spreadsheet by an administrator. A random 10% sample of data will be checked for accuracy.

The qualitative interview will be audio-taped or digitally recorded. Interviews will be transcribed by an administrator, omitting any information that may compromise confidentiality. All audio-tapes will then be destroyed and all electronic transcripts kept on a password protected computer. These will only be made available to those within the research team and any identifiers within the transcripts will be removed so that the data cannot be traced back to the participants. All personal data is stored on an encrypted drive, and links to personal information are available only to research investigators and trial coordinator. Consent forms and questionnaire data are double-entered and stored in locked filing cabinets in a secure site at National University of Singapore.

## Discussion

Worldwide, the shift within health services to a more patient centered approach has underlined the necessity of engaging people in their own health. In long term conditions this shift has the potential of providing large benefits to patients and to improve clinical outcomes. Patients on dialysis are required to make a number of major lifestyle changes and the NKF-NUS self management intervention has been designed to empower patients to take control of their condition.

The trial will evaluate the effectiveness of a brief self management intervention delivered in groups of established hemodialysis patients to improve biological markers of adherence and psychosocial functioning compared to standard care.

Previous research indicates the effectiveness of psychological interventions in clinical and psychosocial outcomes [35-37] but has methodological limitations related to sample size and short duration of follow up. The current trial is the first to evaluate the effects of a theory driven self management intervention in Singapore, in a program delivered by health care professionals rather than trained psychologists. Given the scaricity of trained psychologists in renal services in Singapore, the adopted approach of training existing renal health care professionals already involved in the care of people on hemodialysis maximises potential feasibility of the intergration and delivery in the NKF centres across Singapore. The intervention is fully manualized and supported by a training course ensuring that it can be replicated in a standadized form.

The trial design addresses weaknesses of previous research by use of an adequate sample size to detect clinically significant changes in biochemical markers, recruitment of a sufficiently large and representative haemodialysis sample, definition of a feasible theory based intervention to support treatment adherence, and careful assessment of both clinical and psychological endpoints in order to evaluate whether effects (if any) are sustained over time. Inclusion of multiple dependent variables allows us to assess the broader impact on the intervention including both hard end points as well as patient reported outcomes. A further strength of the trial design is the inclusion of measures that explore the extent to which the intervention impacts upon psychological processes and how these processes act as mediating variables. It will be possible to explore whether the intervention influences beliefs and cognitions (self efficacy expectancies). It will also be possible to examine whether differences in clinical outcomes and behaviours are due to intervention effect on beliefs and cognitions.

The current trial does however pose a number of challenges, perhaps most notably related to the potential of recruitment bias. We have purposefully selected NKF community dialysis centres across Singapore to ensure geographical representation, however, certain dialysis centres were not approached due to the lack of facilities to host the intervention or distance from other dialysis centres with such facilities. Although it would have been preferable to randomly select units among the 24 NKF centres, this was not deemed feasible in this pragmatic trial. As no differences in sociodemographic or ethnic composition of patients across centres/residents in different regions in singapore is expected due to implementation of hosing policies to ensure adequate representation of ethnic groups is public housing projects [59], we feel that this approach will not introduce bias. We will however examine statistically any differences between participating and non participating centres as well as between dialysis shifts in the participating centres. Active steps will be taken to reduce a variety of potential biases through the use of randomisation procedures.

The trial is mainly generalisable to patients who are willing to participate and be randomised and to patients who can competently converse into either English, Mandarin and/or Malay. Patients preferred language of communication will be taken into consideration when forming groups but there are no resources to allow delivery of intervention in patients who only speak dialects (e.g. Hokkien, Teochew) or Tamil. Exclusion of these patients is therefore unavoidable. Participants excluded due to language barriers and control participants will have an opportunity to participate in the program at the end of the trial.

## Competing interests

The authors declare that they have no competing interests.

## Authors' contributions

KG conceived and designed the research and wrote the manuscript. HJ, SPN assisted with the study design and writing of the manuscript. KG drafted the manuscript, and all authors provided critical revisions and have approved the final manuscript.

## Pre-publication history

The pre-publication history for this paper can be accessed here:

http://www.biomedcentral.com/1471-2369/12/4/prepub
